# Seed Shattering: A Trait of Evolutionary Importance in Plants

**DOI:** 10.3389/fpls.2021.657773

**Published:** 2021-06-16

**Authors:** Aniruddha Maity, Amrit Lamichaney, Dinesh Chandra Joshi, Ali Bajwa, Nithya Subramanian, Michael Walsh, Muthukumar Bagavathiannan

**Affiliations:** ^1^Department of Soil and Crop Sciences, Texas A&M University, College Station, TX, United States; ^2^Seed Technology Division, ICAR-Indian Grassland and Fodder Research Institute, Jhansi, India; ^3^Division of Crop Improvement, ICAR-Indian Institute of Pulses Research, Kanpur, India; ^4^Division of Crop Improvement, ICAR-Vivekananda Parvatiya Krishi Anusandhan Sansthan, Almora, India; ^5^Weed Research Unit, New South Wales Department of Primary Industries, Wagga Wagga, NSW, Australia; ^6^Sydney Institute of Agriculture, The University of Sydney, Sydney, NSW, Australia

**Keywords:** weed seed dispersal, seedbank, harvest weed seed control, weed evolutionary adaptation, crop improvement

## Abstract

Seed shattering refers to the natural shedding of seeds when they ripe, a phenomenon typically observed in wild and weedy plant species. The timing and extent of this phenomenon varies considerably among plant species. Seed shattering is primarily a genetically controlled trait; however, it is significantly influenced by environmental conditions, management practices and their interactions, especially in agro-ecosystems. This trait is undesirable in domesticated crops where consistent efforts have been made to minimize it through conventional and molecular breeding approaches. However, this evolutionary trait serves as an important fitness and survival mechanism for most weeds that utilize it to ensure efficient dispersal of their seeds, paving the way for persistent soil seedbank development and sustained future populations. Weeds have continuously evolved variations in seed shattering as an adaptation under changing management regimes. High seed retention is common in many cropping weeds where weed maturity coincides with crop harvest, facilitating seed dispersal through harvesting operations, though some weeds have notoriously high seed shattering before crop harvest. However, high seed retention in some of the most problematic agricultural weed species such as annual ryegrass (*Lolium rigidum*), wild radish (*Raphanus raphanistrum*), and weedy amaranths (*Amaranthu*s spp.) provides an opportunity to implement innovative weed management approaches such as harvest weed seed control, which aims at capturing and destroying weed seeds retained at crop harvest. The integration of such management options with other practices is important to avoid the rapid evolution of high seed shattering in target weed species. Advances in genetics and molecular biology have shown promise for reducing seed shattering in important crops, which could be exploited for manipulating seed shattering in weed species. Future research should focus on developing a better understanding of various seed shattering mechanisms in plants in relation to changing climatic and management regimes.

## Introduction

Plants constantly evolve and adapt in the wild, shaped by natural selection (Darwin, 1859; Lenski, 2017). During the domestication of wild species, humans have intervened and accelerated the selection process for desired plant traits through artificial selection (Gregory, 2009). This has led to the loss of several adaptive traits in plants that are vital for persistence under natural conditions (Pickersgill, 2007; Flint-Garcia, 2013). For example, traits such as non-synchronous flowering, non-uniform seed maturity, seed shattering and seed dormancy are all important traits for wild plant populations in natural environments (Kantar et al., 2017). These traits allow wild plants to germinate, grow and reproduce under conditions that are conducive to their growth and development (Pickersgill, 2007). Among these traits, seed shattering, i.e., the capacity of a plant to shed its seeds, is essential for the dispersal and persistence of the offspring in many wild species (Dong and Wang, 2015). Shattering can occur over a period of a few to several days, increasing the chances that a significant proportion of the produced seeds are dispersed away from the mother plants and new niches are occupied (Delouche et al., 2007). Thus, seed shattering minimizes intra-population competition and increases species fitness (Thurber, 2012; Di Vittori et al., 2019).

In domesticated crops, seed shattering is an unfavorable trait due to its detrimental impact on harvestable grain yield (Serebrenik, 2013; Table 1). Domestication has selected for crops with almost no seed shattering ability, especially in those crops grown for grain production (Harlan et al., 1973). Some level of seed shattering is present and even preferred in pasture grasses and legumes as a specialized adaptation that ensures self-seeding and pasture regeneration (Dong and Wang, 2015). Many cultivated crops, if left as “wild populations,” revert to shattering phenotypes through back mutation (endoferality) as evident in wild rice (*Oryza sativa*) (Vigueira et al., 2013, 2019), or through continued introgression (exoferality) as in shattercane (*Sorghum bicolor* ssp. *drummondii*) (Ejeta and Grenier, 2005). This indicates that the shattering habit might be complementary for the persistence of previously domesticated crop species in undisturbed natural ecosystems (Di Vittori et al., 2019).

**TABLE 1:** Seed loss due to shattering documented in cultivated crops.

Family	Crop	Scientific name	Loss due to shattering (%, unless mentioned otherwise)	References
*Poaceae*	Oat	*Avena sativa*	12–50	Clarke, 1981
	Barley (rainfed)	*Hordeum vulgare*	0–34	Platt and Wells, 1949
	Rice	*Oryza sativa*	1–5	Niruntrayakul et al., 2009
			28 g/plant (greenhouse); 61 g/plant (growth chamber)	Thurber et al., 2013
	Dallisgrass	*Paspalum dilatatum*	30	Bennett and Marchbanks, 1969
	Bahiagrass	*Paspalum notatum*	36–50	Correa, 1974
*Brassicaceae*	Indian mustard	*Brassica juncea*	4–7	Gan et al., 2008
	Canola	*Brassica napus*	6	Gulden et al., 2003
			8	Gan et al., 2008
			50	Price et al., 1996
	Yellow mustard	*Sinapis alba*	5	Gan et al., 2008
	Rape mustard	*Brassica rapa*	2	Gan et al., 2008
*Fabaceae*	Chickpea	*Cicer arietinum*	65	Murgia et al., 2017
	Soybean	*Glycine max*	5–10	Davidson, 2014
			21	Tukamuhabwa et al., 2002
			37	Philbrook and Oplinger, 1989
			34–99	Tiwari and Bhatnagar, 1991

Seed shattering is a highly diverse trait in weedy and wild species, e.g., in Italian ryegrass (Maity et al., 2021), influenced by years of selection (Vigueira et al., 2013; Table 2). Shattering of seed and its effective dispersal enable the weeds to survive and persist in natural as well as agricultural landscapes (Thurber, 2012). However, shattering can lead to substantial crop yield loss in commercial agriculture. In this review, the significance of seed shattering in crops and weeds, mechanisms of seed shattering and how different factors influence this important trait are discussed. A snapshot of how the recent developments in plant physiology, genetics and genomics have contributed to our understanding of this complex trait is also presented. The synthesis of knowledge on this important aspect of plant evolutionary biology is beneficial for crop improvement as well as weed management in modern agriculture.

**TABLE 2:** Seed shattering values at crop harvest for major weeds in global cropping systems.

Family	Scientific name	Common name*	Seed shattering (%) prior to main crop harvest**	Country/state or province	References
*Amaranthaceae*	*Amaranthus tuberculatus*	Tall waterhemp	1–5	United States/Nebraska, Missouri, Wisconsin, and Illinois	Schwartz et al., 2016
	*Amaranthus palmeri*	Palmer amaranth	1–5	United States/Arkansas, Tennessee, Illinois, Missouri, and Nebraska	Schwartz et al., 2016
			10	United States/Puerto Rico	Green et al., 2016
	*Amaranthus retroflexus*	Redroot pigweed	48	United States/Virginia	Haring et al., 2017
			44	Canada/Alberta	Beckie et al., 2017
*Amaranthaceae*	*Chenopodium album*	Common lambsquarters	10	Alberta, Canada	Beckie et al., 2017
			9	United States/Virginia	Haring et al., 2017
			50	United States/Minnesota	Forcella et al., 1996
	*Bassia scoparia*	Kochia	0	Canada/Saskatchewan	Burton et al., 2017
			0	Canada/Alberta and Saskatchewan	Tidemann et al., 2017
			0	Canada/Saskatchewan	Burton et al., 2017
			0	Canada/Alberta	Beckie et al., 2017
*Asteraceae*	*Ambrosia artemisiifolia*	Common ragweed	38	United States/Virginia	Haring et al., 2017
	*Ambrosia trifida*	Giant ragweed	20	United States/Minnesota	Goplen et al., 2016
			40	United States/Virginia	Haring et al., 2017
	*Conyza bonariensis*	Horseweed/flaxleaf fleabane	7–81	Australia/Queensland and New South Wales	Widderick et al., 2014
	*Sonchus asper*	Prickly sowthistle	92	Canada/Alberta	Beckie et al., 2017
	*Sonchus oleraceus*	Common sowthistle	46–62	Australia/Queensland and New South Wales	Widderick et al., 2014
*Brassicaceae*	*Brassica napus*	Canola	2	Canada/Alberta and Saskatchewan	Tidemann et al., 2017
	*Raphanus raphanistrum*	Wild radish	1	Australia/Western Australia Canada/Alberta	Burton et al., 2017; Walsh and Powles, 2014
			10	Australia/Western Australia	Walsh and Powles, 2014
	*Rapistrun rugosum*	Turnip weed	0–81	Australia/Queensland and New South Wales	Widderick et al., 2014
	*Sinapis arvensis*	Wild mustard	30	Canada/Saskatchewan Canada/Alberta	Beckie et al., 2017; Burton et al., 2017
			100	United States/Minnesota	Forcella et al., 1996
	*Sisymbrium thellungii*	African turnip weed	0	Australia/Queensland and New South Wales	Widderick et al., 2014
*Malvaceae*	*Hibiscus trionum*	Flower of an hour/Bladder ketmia	45–79	Australia/Queensland and New South Wales	Widderick et al., 2014
	*Malva neglecta*	Buttonweed	0	Canada/Alberta	Beckie et al., 2017
*Poaceae*	*Aegilops cylindrica*	Jointed goatgrass	30	Australia/Western Australia	Walsh and Powles, 2014
			25	United States/Colorado	Soni et al., 2020
	*Alopecurus myosuroides*	Slender meadow foxtail	40–90	United Kingdom	Walsh et al., 2018
	*Avena fatua*	Wild oat	61	Canada/Alberta	Beckie et al., 2017
			22–30	Canada/Saskatchewan	Burton et al., 2017
			16–31	Western Australia, Australia	Walsh and Powles, 2014; Widderick et al., 2014
	*Avena fatua; Avena sterilis*	Wild oat	80–96	Spain and United Kingdom	Barroso et al., 2006
	*Bromus tectorum*	Cheatgrass/downy brome	33	Australia/Western Australia	Walsh and Powles, 2014
			25	United States/Colorado	Soni et al., 2020
	*Chloris virgata*	Rhodesgrass	29–53	Australia/Queensland and New South Wales	Widderick et al., 2014
	*Digitaria sanguinalis*	Large crabgrass	77	United States/Virginia	Haring et al., 2017
	*Echinochloa colona*	Jungle rice	5–91	Australia/Queensland and New South Wales	Widderick et al., 2014
			59–68	United States/Arkansas	Schwartz-Lazaro et al., 2017
			67	United States/Puerto Rico	Green et al., 2016
	*Lolium rigidum*	Rigid ryegrass	15	Australia/Western Australia	Walsh and Powles, 2014
			15	Spain/Catalonia	Blanco-Moreno et al., 2004
	*Lolium perenne* ssp. *multiflorum*	Italian ryegrass	4.8–54	United States/Texas	Maity et al., 2021
	*Oryza sativa*	Red rice	15–87	United States/Arkansas	Burgos et al., 2014
	*Secale cereale*	Feral rye	25	United States/Colorado	Soni et al., 2020
			10	Spain/Catalonia	Blanco-Moreno et al., 2004
	*Setaria faberi*	Giant foxtail	68	United States/Virginia	Haring et al., 2017
			60	United States/Minnesota	Forcella et al., 1996
	*Setaria viridis*	Green foxtail	6	Canada/Saskatchewan; Canada/Alberta	Beckie et al., 2017; Burton et al., 2016, 2017
			80	United States/Virginia	Haring et al., 2017
	*Sorghum halepense*	Johnsongrass	0–50	United States/Texas	Young, 2021
			0–30	United States/Arkansas	Young, 2021
*Polygonaceae*	*Polygonum convolvulus*	Black bindweed	18	Canada/Saskatchewan	Burton et al., 2017
*Rubiaceae*	*Galium* spp.	Cleavers	26	Canada/Alberta	Beckie et al., 2017
	*Galium spurium* + *aparine*	Cleavers	2–4	Canada/Saskatchewan	Burton et al., 2017

*Common names are according to the list approved by Weed Science Society of America (WSSA). **A substantial portion of this data was obtained from Walsh et al. (2018).

## Factors Controlling Seed Shattering in Plants

Seed shattering in plants is regulated by complex physiological and genetic mechanisms (Zhao et al., 2019), in conjunction with environmental factors. Some of these mechanisms are fairly well understood in domesticated crops, whereas little is known for most wild and weedy species.

### Physiological Control

The first step in seed or pod shattering is the formation of an abscission layer at the point where the seeds or pods are attached to the plants. Though the fundamental mechanism of abscission is the same for many crops, it varies with the type of tissue, as it may be the spikelet in cereals or a pod in legumes (Dong and Wang, 2015). Two main series of events occur during the process of abscission: the first is the disintegration of the entire or a portion of the cell wall as a result of biochemical changes, which is then followed by the mechanical tearing of the abscission layer (Pfeiffer, 1928). In the first event, the cells in abscission layers become elongated and eventually collapse after plasmolysis. In the second event, a sudden disruption of the abscission cells occurs due to enzymatic deterioration, resulting in the tearing of the abscission layer (Pfeiffer, 1928).

**FIGURE 1:**
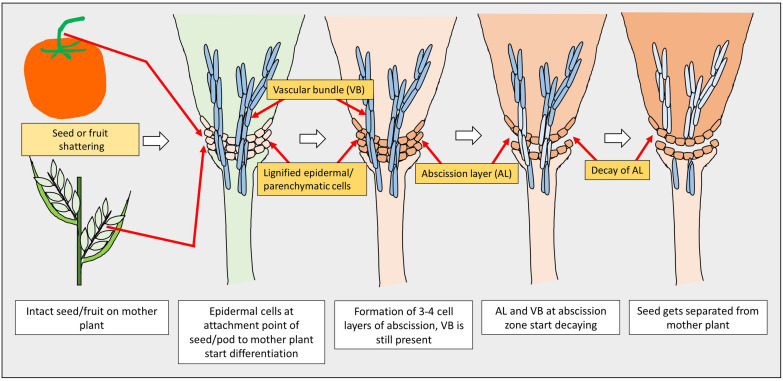
Diagram showing stages of seed shattering in monocot and dicot plants.

A model of seed shattering in monocot or fruit dehiscence in dicot is presented in Figure 1. In monocots, seed shattering is triggered by the formation of an abscission layer at the attachment point between the lemma and pedicel by cell wall thickening and lignification (Harlan and DeWet, 1965; Elgersma et al., 1988; Fuller and Allaby, 2009). Swelling and dissolving of the middle lamella between adjacent cell walls in the abscission layer allows for grain release (Htun et al., 2014). The structure and stage of formation and the anatomical location of the abscission layer may vary among plant species. In rice, development of an abscission layer between the spikelet and rachilla, followed by its degradation leads to seed shattering (Zheng et al., 2007; Fuller and Qin, 2008). Examination of the spikelet bases between domesticated rice and the wild shattering types revealed that domesticated spikelet bases are characterized by a dimpled appearance and possess less symmetrical scars, whereas the wild types had a smooth scar with a straight profile at the spikelet bases (Li et al., 2006; Fuller et al., 2009). In *Lolium* spp., the abscission layer is present at the attachment point of lemma and palea to the rachilla (Elgersma et al., 1988). The abscission layer is easily identifiable as the cells present in it are smaller than the parenchymatous cells in the rachilla. In perennial ryegrass (*Lolium perenne* L.), this layer usually consists of 4–8 cell layers (Elgersma et al., 1988). In bahiagrass (*Paspalum notatum* Fluegge), cells in the abscission layer were larger, more prominent and present in five to seven layers. The dimension of the dehiscence zone or abscission layer shows positive correlation with shattering resistance, as reported by Child et al. (2003) in *Brassica napus*. These cells eventually lost their wall, leading to shattering. In wild and weedy species, development of the abscission layer has been shown to occur at a much faster rate compared to their cultivated counterparts (Li et al., 2006). In wild rice, the abscission layer forms before flowering and begins degradation during the course of flowering, whereas in cultivated rice the abscission layers remain intact and show no sign of degradation even after flowering (Carrie et al., 2011). In dalliagrass (*Paspalum dilatatum* Poir), the abscission layer was identifiable between early booting and booting stages (Burson et al., 1978).

**FIGURE 2:**
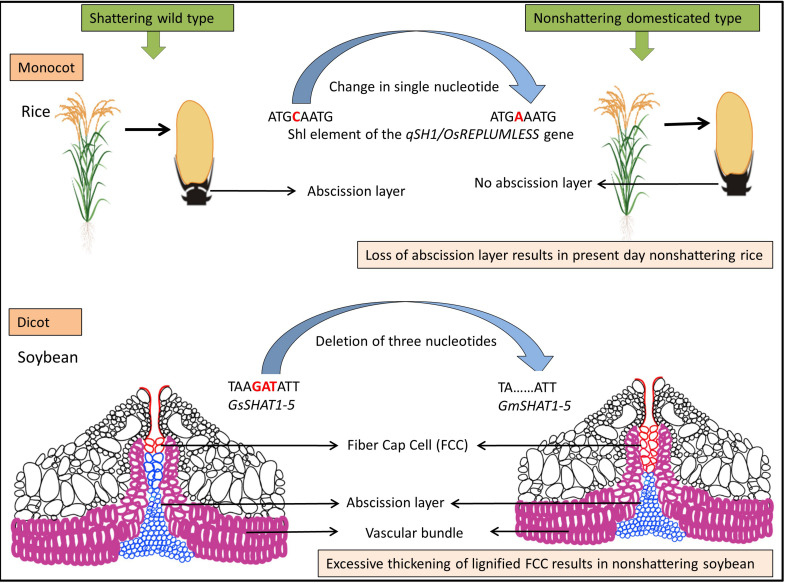
Model for evolution of shattering resistance in cultivated rice [modified from Gasser and Simon (2011)] and dehiscence resistance in cultivated soybean [modified from Dong et al. (2014)].

In dicots, studies on the mechanisms responsible for seed shattering (more appropriately, dehiscence of pod in legumes and siliqua in crucifers) are meager compared to that of monocots (Lin et al., 2012; Dong and Wang, 2015). Most relevant studies on pod dehiscence (development of abscission zones along the pod valve margin) have been conducted in soybean and French bean (*Phaseolus vulgaris* L.) (Romkaew et al., 2008; Dong et al., 2014; Murgia et al., 2017). Dehiscence in less domesticated crops begins long before the actual dehiscence, sometimes as early as the fertilization of the ovule (Ferrándiz et al., 1999). Pod dehiscence in dicots is induced by the formation of a specific dehiscence (or abscission) zone along the pod (Dong and Wang, 2015; Figure 2). The cells at the abscission zone start differentiating into lignified and separation layers during pod development, which then auto-degrade before pod dehiscence (Seymour et al., 2013). Lignification is a complex process involving the deposition of lignins on the extracellular polysaccharidic matrix (Ros, 1997), and a higher degree of lignification in the abscission layer cells indicates more shattering (Lee et al., 2018). The degree of lignification of the inner layer of the pod wall determined the extent of pod dehiscence in common bean (Murgia et al., 2017) and soybean (Funatsuki et al., 2014). In addition to lignin, other main fibers of the plant secondary cell wall such as cellulose and hemi-cellulose, alone or in combination provide strength and structural integrity to cell walls, which directly affect shattering (Baucher et al., 1998). Suanum et al. (2016) observed in yardlong bean (*Vigna unguiculata* ssp. *sesquipedalis*) and wild cowpea [*Vigna unguiculata* (L.) Walp.] that cellulose, hemi-cellulose and lignin contents in pods are highly correlated with pod dehiscence. The non-shattering genotypes have several layers of thickened fiber cap cells compared to the shattering types (Figure 2).

Seed shattering in monocots and dicots is determined by a complex plant signaling network involving hormones (Vivian-Smith and Koltunow, 1999). Thickening, swelling, and dissolving of the cell layers in the abscission zones across plant parts are accomplished by up- and down-regulations of certain gene(s) triggering the production and activity of specific enzyme(s) and plant hormone(s). An increase in β-1,4-glucanase or cellulase activity has been observed during pod dehiscence in canola (*B. napus*) (Roberts et al., 2002), whereas increasing polygalacturonase activity is correlated with shedding of fruits in oil palm (*Elaeis guineensis*) (Henderson et al., 2001). A number of proteins such as expansin and chitinase (a Pathogenesis-Related Protein) are reported to directly influence the abscission process in various plant parts across multiple plant species (reviewed in Roberts et al., 2002). In addition to gibberellins (GA), abscisic acid (ABA) and cytokinin (CYT), ethylene (ETH) and auxin (IAA) concentrations in the abscission or dehiscence zones are also known to play a major role in determining seed shattering or pod dehiscence (Addicott, 1970; González-Carranza et al., 1998). RNA-sequencing and expression analysis show that the specific ABA-responsive 9-*cis-epoxycarotenoid dioxygenase* (NCED) gene, a key gene for ABA biosynthesis, and ABA concentration increase prior to and during abscission process and show a potential signal transduction network among the plant hormones involved in seed shattering (Lang et al., 2021). However, several studies suggest ETH as the primary regulator of seed shattering and ABA’s critical role depends on its interaction with IAA and ETH, suggesting an intermediary role of ABA in organ abscission (Marciniak et al., 2018). Cellulase activity shows a high positive correlation with the level of IAA, leading to rapid abscission (Chauvaux et al., 1997). Specifically, high concentration of auxins negatively influences seed shattering. Application of IAA on mature silique retarded the cellulase activity and pod dehiscence (Chauvaux et al., 1997), whereas ETH promoted the formation of the dehiscence zone (Ferrándiz, 2002). However, depending on the species, stage of application, and biochemical form, IAA can accelerate the abscission process (Addicott and Lynch, 1951). In *Arabidopsis*, studies have established correlation between dehiscence zone development and low levels of IAA (Heisler et al., 2001; Martinez and Vera, 2009). The commonly accepted model of abscission induction in plant organs involves the decrease of IAA levels and increase of ABA, GA, and ETH levels (Meir et al., 2010; Nakano and Ito, 2013; Marciniak et al., 2018).

### Genetic Control

**FIGURE 3:**
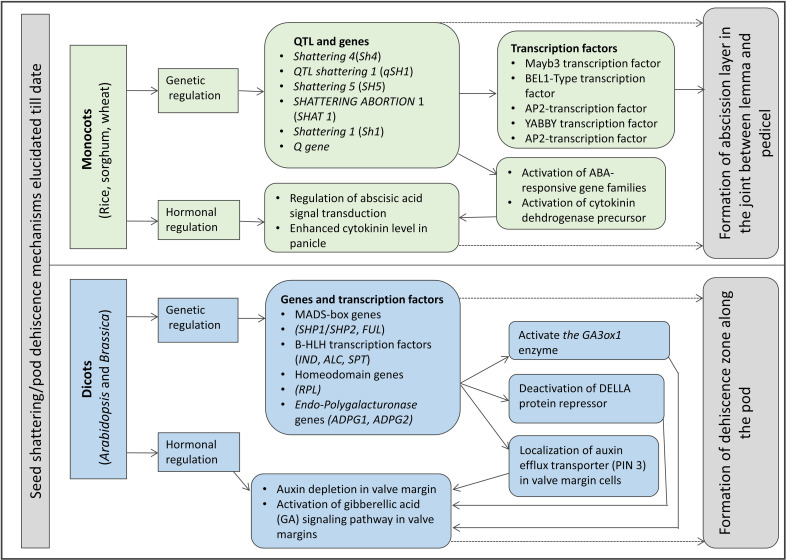
A schematic of genetic mechanisms underlying pod and seed shattering in crop plants.

The genetic mechanisms underlying seed shattering are regulated by a complex network of genes and their interactions (Dong and Wang, 2015; Figure 3). Various investigations aiming at deciphering the genetic mechanisms of seed shattering have indicated the parallel evolution of the non-shattering trait in cereals (Paterson et al., 1995; Konishi et al., 2006; Li et al., 2006; Lin et al., 2012; Tang et al., 2013; Fu et al., 2018). Reports suggest that seed shattering is usually a dominant trait, governed by few, recessive genes across species (Ladizinsky, 1985); e.g., four in rice (Tang and Morishima, 1989), two in common and durum wheat (Love and Craig, 1919), one is cowpea (Aliboh et al., 1997), and two in turnip rape (Mongkolporn et al., 2003; Table 3). The major seed shattering gene in sorghum (*Sh1*; that encodes a YABBY transcription factor) and its orthologs in rice induce seed shattering through one common mechanism, i.e., formation of an abscission layer between the pedicel and spikelet (Lin et al., 2012; Li et al., 2019). The loss-of-function mutation in these genes is independently selected for non-shattering in domesticated sorghum, rice (Lv et al., 2018) and corn (*Zea mays* L.) (Paterson et al., 1995; Lin et al., 2012). Konishi et al. (2006) reported that a single nucleotide change resulted in a non-shattering trait in domestic rice. In soybean, three nucleotides in the *GsSHAT1-5* gene lead to a non-shattering type (Dong et al., 2014; Figure 3).

**TABLE 3:** Inheritance pattern of seed shattering trait in selected plant species.

Plant species	Scenarios	Genetic control/Inheritance pattern	References
Rice	*Oryza sativa*, weedy strains X cultivated strains	Four genes with segregation patterns ranging from monogenic to continuous, depending on the crosses	Tang and Morishima, 1989
Common and durum wheat	*Triticum vulgare* X *T. durum*	One gene with dominant gene action, shattering is dominant to non-shattering	Love and Craig, 1919
Einkorn wheat	*Triticum monococcum* X *T. boeoticum*	Two recessive genes with additive action, shattering is dominant to non-shattering	Sharma and Waines, 1980
Ryegrass	*Lolium temulentum* X *L. persicum*	Two recessive genes with additive action, shattering is dominant to non-shattering	Senda et al., 2006
Foxtail millet	*Setaria viridis* X *S. italica*	Two genes with additive action, hybrids with 0 or 1 allele from the shedding parent show no shedding, but with 2 or more alleles show shedding	Darmency and Pernes, 1987
Pearl millet	*Pennisetum mollissimum* X *P. glaucum*	One gene with dominant gene action, shattering is dominant to non-shattering	Poncet et al., 1998
Buckwheat	*Fagopyrum homotropicum* X *F. esculentum*	Three complementary dominant genes	Wang et al., 2005
Cowpea	*Vigna unguiculata*, wild X cultivated	Monogenic dominance of pod shattering over non-shattering	Aliboh et al., 1997
Turnip rape	*Brassica rapa*, shatter-susceptible X shatter-resistant	Two recessive major genes with a dominant epistasis effect	Mongkolporn et al., 2003

The genus *Medicago* is known for its pod coiling mechanism of seed dispersal, which is highly correlated with the increased valve margin lignification mechanism of the members of *Brassicaceae* (Fourquin et al., 2013). Genetic analysis elucidated that the pod coiling mechanism is governed by a minor protein sequence of *SHATTERPROOF* (SHP) orthologs (Ferrándiz and Fourquin, 2014). In rice, *qSH1* [the major Quantitative Trait Loci (QTL) on chromosome 1 that controls seed shattering] is required for the formation of the abscission layer in the pedicel. It encodes a BEL-1 type homeobox transcription factor that is homologous to the *RPL* transcription factor of *Arabidopsis*, which is required for the development of replum cells in silique wall margin (Konishi et al., 2006). Suanum et al. (2016) indicated that the major QTL for the fibers such as cellulose, hemi-cellulose and lignin in pods of yardlong bean and wild cowpea are co-located with the major pod shattering QTL. Furthermore, over-expression analysis of the *NAC* and *SHAT-5* genes in soybean revealed that shattering-resistant lines had increased biosynthesis of a secondary wall that resulted in thickening of lignified fiber cap cells (Dong et al., 2014). These findings suggest that a unique convergent mechanism is involved in seed shattering across distantly related genera.

In recent years, bi-parental mapping and genome-wide approaches have enabled dissection of the complex genetic control of seed shattering (Table 4). Specific genes and transcription factors regulating morphological and anatomical mechanisms governing seed shattering have been identified in both monocot and dicot species (Table 5). Various studies have found QTL explaining up to 50% of phenotypic variance for seed shattering (Funatsuki et al., 2006; Subudhi et al., 2014; Table 4). Recently, fourteen candidate gene derived polymorphic EST-SSR markers specific for abscission zone development and seed shattering were developed in *Elymus nutans* (Zhao et al., 2019). Though the abscission layer formation in plants appears to be controlled by a few major genes (McWilliam, 1980), the final quantum of seed shattering is a highly environment-dependent event, which varies significantly among cultivars, geography and seasons (Konishi et al., 2006), suggesting that seed shattering is a complex, polygenic trait (Zhou et al., 2012).

**TABLE 4:** The specific Quantitative Trait Locus/Loci (QTL) reported to influence seed/pod shattering in food crops.

Crop	Mapping Population	Markers	Identified QTL	Linkage group	Phenotypic variation	References
**Monocot**						
Hybrid *Elymus*	164 backcross progenies derived from creeping × basin wildrye hybrid and a true creeping wildrye tester	-	One pleiotropic QTL	6	43.1%	Larson and Kellogg, 2009
Rice	120 DH lines derived from a cross between Cheongchenong × Nagdong	217 SSR markers	3 QTL for pulling strength and 4 QTL for bending strength	1,2,4,6,9,10	5–14%	Lee et al., 2016
	198 F_7:8_ RILs derived from the cross Bengal × PSRR-1 and 174 F_8:9_ RILs derived from the cross Cypress × PSRR-1	SSR markers	Two QTL were consistent across the populations	4 and 10	61.9%	Subudhi et al., 2014
	CSSLs and NILs of Japonica rice landrace Jiucaiquing in IR-26 background	192 SSR markers	Four QTL	1, 3, 6 and 11	-	Cheng et al., 2016
**Dicot**						
Abyssinian Cabbage	229 F_2_ lines derived from BC 73526 × BC 73524	6,464 DArT-Seq Markers	Five QTL	B1, B3, B8 and C5	3.75–5.27%	Raman et al., 2017
Azuki bean	188 F_2_ lines derived from a cross between JP 110658 × JP 109685	316 SSR markers	Two QTL	4 and 9	6.4–18.2%	Kaga et al., 2008
Canola	126 DH lines derived from BLN2762 × Surpass 400	DArT-Seq markers	Twelve QTL	3, 4, 6, 7,8,9	57%	Raman et al., 2014
	Unstructured diversity panel of 143 accessions and two structured populations (96 DH lines and 124 F_2_ progenies)	-	Two QTL consistently detected across the populations and environments	A06 and A09	-	Liu et al., 2016
Cowpea	159 RILs derived from a cross between 524B × 219-01	202 SSR markers	Four QTL	1 to 10	6.4 to 17.2%	Andargie et al., 2011
	215 RILs derived from a cross between IT99K-573-1-1 × TVNu-1158	51,128 SNPs	Two QTL	3 and 5	68%	Lo et al., 2018
Soybean	104 RILs derived from the cross Toyomusume × Hayahikari and 96 F_2_ lines derived from the cross Toyomusume × HC1-F7-57	178 SSR markers	One major QTL detected across the populations	10	50%	Funatsuki et al., 2006

**TABLE 5:** Genes and transcription factors reported to control seed shattering in a number of plant species.

Plant species	Gene/Transcription factor	Mechanism	Nature of allele for breeding	References
Arabidopsis	*SHP1* and *SHP 2*	Lignification of silique valve margin and the adjacent cells	Loss of function	Liljegren et al., 2000
	*FUL*	Lignification of silique valve margin and the adjacent cells	Ectopic expression	Liljegren et al., 2000
	*ALC*	Encode a protein related to the myc/bHLH family of transcription factors which promotes separation of the valve cells from the replum	Loss of function	Rajani and Sundaresan, 2001
	*RPL*	Encodes a homeodomain protein that prevents development of replum cells into silique valve margin	Loss of function	Roeder et al., 2003
	*STK* or *AGL 11*	Promotes the proper development of funicules by forming a clear abscission zone	Loss of function	Pinyopich et al., 2003
	*IND*	Encodes a basic helix-loop-helix protein involved in patterning of the fruit cell types required for seed dispersal	Loss of function	Liljegren et al., 2004
	*NST 1 and NST 3*	Encodes protein which promote secondary walls synthesis in valve margins are required for dehiscence	Loss of function	Mitsuda and Ohme-Takagi, 2008
Rice	*SHAT 1*	Encodes a transcription factor (*APETALA*_2_) required for the formation of abscission zone pedicel and spikelet	Loss of function	Zhou et al., 2012
	*SSH1*	Encodes an *APETALA*_2_-like transcription factor SUPERNUMERARY BRACT required for the formation of abscission zone pedicel and spikelet	Loss of function	Jiang et al., 2019
	*qCSS3*	Improves seed shattering resistance	Loss of function	Tsujimura et al., 2019
	*OsGRF*_4_	Improves seed shattering resistance by differential abscission zone formation	High expression	Sun et al., 2016
Sorghum	*Sh 1*	Encodes a transcription factor YAABY required for the formation of abscission zone	Loss of function	Olsen, 2012
	*SpWRKY*	Suppress the downstream cell wall biosynthesis genes to allow deposition of lignin that initiates abscission zone formation in the seed pedicel junction	Loss of function	Tang et al., 2013
Soybean	*Pdh1*	Encodes a dirigent-like protein which promotes pod dehiscence by increasing the torsion of dried pod walls under low humidity	Loss of function	Funatsuki et al., 2014
	*SHAT1-5*	promotes the significant thickening of fiber cap cells	Over expression	Dong et al., 2014
Wheat	*Q*	encodes a member of AP2-family transcription factor which confers the free threshing character	Ectopic expression	Simons et al., 2006

### Environmental Control

Seed shattering in plants is strongly influenced by genotype × environmental (G × E) interactions (Liu et al., 2016). Though seed shattering is genetically controlled, the degree of shattering is influenced by the environmental conditions that plants experience during their growth (Tiwari and Bhatnagar, 1989; Thurber, 2012). Specifically, temperature, humidity and moisture appear to influence seed shattering. High temperature conditions are shown to increase seed shattering in rice (Ji et al., 2006; Thurber et al., 2010), chickpea (*Cicer arietinum* L.) (Van Gastel et al., 2007), birdsfoot trefoil (*Lotus corniculatus* L.) (Garcia-Diaz and Steiner, 2000), and soybean (Tsuchiya, 1987). Low humidity in canola (*Brassica napus* L.) (Tsuchiya, 1987; Gan et al., 2008) and soybean (Tiwari and Bhatnagar, 1989), high precipitation in canola (Vera et al., 2007) and soybean (Tiwari and Bhatnagar, 1989), and high wind in oilseed crops (Vera et al., 2007; Gan et al., 2008) have been shown to increase seed shattering. Conditions such as low humidity, high temperature, rapid temperature changes, wetting and drying, etc., which reduce the level of seed/pod moisture content, may ultimately induce pod shattering in soybean (Buckovic, 1952; Tsuchiya, 1987). The rate of moisture loss differs between two adjacent tissue layers of the abscission zone at the sutures, increasing the tension between the individual layers, eventually resulting in separation of the two valves of the pod leading to seed shattering (Buckovic, 1952). Some environmental conditions indirectly alter seed shattering window, by influencing physiological maturity. For instance, high temperature conditions during reproductive transition can induce early flowering (Maity et al., 2012; Pope et al., 2013), which can in turn result in early seed or fruit shedding. The enzymatic and biochemical mechanisms (discussed in section “Genetic Control”) that govern seed development and shattering are reported to be highly sensitive to environmental stresses (reviewed in Maity et al., 2016). For example, cellulase (Wang et al., 2011) and polygalacturonase (Yoshida et al., 1984), two important enzymes associated with seed shattering, are highly responsive to temperature stress. Consequently, seed shattering is influenced by environmental factors influencing at cellular levels, leading to visible phenotypic changes.

Plant acclimatization to different environments can also play a significant role on the extent of seed shattering. For example, Burton et al. (2017) indicated that seed shattering is less in early maturing crops. Elgersma et al. (1988) reported that crops with erect growth habit are prone to shedding seeds prior to harvest, because in a crop with prostrate growth habit, the seed heads are somewhat protected against wind. Plant morphological characteristics such as vascular structure, pod structure or vascular bundle size can influence seed shattering (Summers et al., 2003). Further, seed moisture content can also affect pod shattering, as shown in chickpea by Margheim et al. (2004).

## Seed Shattering in Domesticated Crops

Seed shattering (or pod dehiscence in legumes, fruit shedding or spikelet shedding of grass spikes/panicles) is the first step in the process of seed dispersal (Harlan et al., 1973; Zhou et al., 2012). Seed shattering is an important weedy trait, and is a key trait that differentiates cultivated and wild plants (Onishim et al., 2007). In addition to causing grain yield loss, presence of substantial seed shattering in feral and de-domesticated populations of cultivated types can be a concern as they favor dispersal. Repeated use of weedy and wild relatives of crop cultivars as genetic resources for improving various traits in crop breeding program might have led to introgression of seed shattering gene(s) or QTL(s) in cultivated types, leading to rapid appearance of ferality and/or de-domestication (Vigueira et al., 2013). In crops, high seed retention or reduced seed shattering has always been a high priority (Hillman and Davies, 1990, 1999). This trait has been selected independently in several species across diverse geographical regions (Di Vittori et al., 2019), and is highly influenced by environmental conditions (Ji et al., 2006; Thurber, 2012).

During domestication, some plant traits have been lost, altered or accumulated over many generations such that cultivated types are genetically distinguishable from their wild ancestors. These collective changes are known as domestication syndrome (Hawkes, 1983; Hammer, 1984; Harlan, 1992). Reduced seed shattering, altered seed dispersal mechanisms, low dormancy, early maturity, decrease in seed phenol or tannin content, thick seed coat, alteration in seed size, seed color, etc. are some notable traits associated with domestication syndrome (Doebley et al., 2006). An analysis by Meyer et al. (2012) on the occurrence of important domestication syndrome traits in 203 crops found that selection for seed retention or reduced seed shattering started about 10,000 years ago. Since the beginning of domestication, seed retention has been considered a valuable trait, and consequently selection has been made against shattering over the years by farmers and plant breeders. However, seed shattering still exists in cultivated crops, contributing significantly to yield losses (Serebrenik, 2013; Table 1). Though modern crop cultivars have substantially low inherent and environment-induced (wind, rain, etc.) seed shattering, this trait could not be completely eliminated in several crops (Gepts and Debouck, 1991; Li et al., 2006; Di Vittori et al., 2019). For instance, weedy amaranths (e.g., Palmer amaranth) exhibit seed shattering (e.g., Schwartz-Lazaro et al., 2017), whereas the grain amaranths are bred as non-shattering types (Brenner, 2002). This is true for many other genera such as *Helianthus* (Burke et al., 2002) and *Linum* (Fu, 2011).

The extent of seed shattering highly varies across domesticated crop species, as influenced by the selection intensity imposed during domestication (Dong and Wang, 2015). Seed shattering has been widely studied in some plant families such as *Brassicaceae* [e.g., *Brassica napus* (Gulden et al., 2003); *Arabidopsis thaliana* (L.) Heynh. (Di Vittori et al., 2019)], *Poaceae* [e.g., rice; *Oryza sativa* (L.) (Vigueira et al., 2013)], and *Fabaceae* [e.g., French bean (Dong et al., 2014)]. Species with high fecundity levels tend to shatter a higher number of seeds (Boelt and Studer, 2010). Moreover, small-seeded biotypes are known to shatter more seed compared to large-seeded types (Sun et al., 2016). Some crop species such as range/pasture species are bred to retain some level of seed shattering to maintain a persistent seedbank for natural regeneration in long-term pastures. Moreover, seed retention is not considered a primary breeding target for forage species because it is suggested that the photosynthates required for high seed retention would reduce the volume of biomass production (Boelt and Studer, 2010; Huff, 2010; Humphreys et al., 2010), though there are exceptions (Griffiths, 1965; Saeidnia et al., 2017). However, when forage grass species are grown as annual pastures, seed shattering can be problematic since only a short pasture phase (1 to 2 years) is required or seed is to be harvested (Lemke et al., 2003). Meyer et al. (2012) estimated that seed shattering occurs at an average of 16% across different crops.

## Seed Shattering in Wild and Weedy Species

Weeds have a tremendous ability to adapt to various selection pressures in agroecosystems (Charbonneau et al., 2018; Huang et al., 2018). Some of the notable adaptive traits in weedy plants include rapid growth habit, short life cycle, efficient seed dispersal and seed dormancy (Baker, 1965). Seed shattering has also been recognized as an essential adaptive trait that favors seed dispersal, seedbank establishment and weediness in many species (Constantin, 1960; Delouche et al., 2007; Burton et al., 2017). Most weeds are prolific seed producers and have the ability to distribute seed shattering over a long duration following physiological maturity (Burton et al., 2017). Seed shattering, however, greatly varies among different weed species, their biotypes and environmental conditions (Table 2). Seed shattering is genetically controlled, but is largely regulated by environmental conditions and agronomic practices (Shirtliffe et al., 2000; Walsh and Powles, 2014).

In arable weeds, seed shattering phenology can be highly variable, and is largely shaped by production practices. In mechanically harvested systems, for example, many annual weed species retain majority of their seeds till crop harvest so that the seed can be harvested and spread across the field by the harvest machinery (Walsh and Powles, 2014), which allows for the persistence of the species in crop fields for years (Shivrain et al., 2010). In many weeds, some level of seed retention at harvest may contaminate harvested crop seed, allowing for long-distance dispersal (Wilson et al., 2016). For example, Chinese sprangletop (*Leptochloa chinensis* L.) in northern Italy was presumed to have been introduced via contaminated rice seed from non-European countries (Benvenuti, 2004). Conversely, weeds may shatter the majority of their seed before crop harvest as an evolutionary adaptation. This adaptation can also be a direct response to harvest weed seed control (HWSC) technology in which all the seeds retained by weeds are captured at crop harvest and destroyed (Walsh et al., 2013; Walsh and Powles, 2014). It is important to note that HWSC is widely adopted only in Australia and the evidence of enhanced seed shattering as an adaptive mechanism against HWSC is still limited (Walsh et al., 2018). In this section, we specifically highlight four arable weed species that are known to exhibit high levels of shattering, to offer valuable insights on the field implications of this trait.

### Shattercane

Shattercane (*Sorghum bicolor*) is a troublesome weed in summer row crops and is a weedy relative of cultivated sorghum (Defelice, 2006; Ohadi et al., 2018). The name shattercane derives from the habit that this race shows profuse seed shattering at physiological seed maturity stage (Defelice, 2006), even with only a slight breeze (Clark and Rosenow, 1992). Individual panicles of shattercane produce about 1,500-2,000 seeds (Roeth et al., 1994; Kegode, 1995), which typically shatter before crop harvest, ensuring seedbank replenishment before they are harvested with the main crop and removed (Dong and Wang, 2015). Kegode (1995) noted that about one-third of all biotypes of shattercane (especially the open-panicle types) drop seed when panicles mature (Kegode, 1995). The shattered seeds can remain viable in the soil seedbank for up to 13 years (Burnside et al., 1997). According to a survey conducted by Roeth et al. (1994) in Nebraska, the top four inches of soil in fields infested with shattercane contained up to 57 million viable seeds per hectare.

### Weedy Rice

Weedy rice (*Oryza sativa* f. *spontanea*) is a common and troublesome weed of cultivated rice (Burgos et al., 2008). Weedy rice is morphologically very diverse and tends to shed seeds from the panicle before the harvest of cultivated rice (Gross et al., 2010; Nadir et al., 2017). Chin et al. (1999) reported a 19–56% seed shattering in weedy rice at 8–15 days after rice flowering in Vietnam. In Italy, Ferrero and Vidotto (1999) documented 65% weedy rice shattering at 30 days after rice flowering. Apart from the common weedy rice, *Oryza rufipogon*, a wild ancestor of cultivated rice which is native to the tropical wetlands of South Asia also shows a high degree of seed shattering (Vigueira et al., 2019). The selection pressure during the course of evolution across the world has resulted in co-evolution of modern non-shattering rice (Li et al., 2006; Di Vittori et al., 2019). However, limited efforts in maintaining the domesticated traits have sometimes culminated in the reversion of domesticated type to wild forms through de-domestication (Vigueira et al., 2013; Kanapeckas et al., 2016). For example, seed shattering in feral weedy rice was acquired during de-domestication (Kanapeckas et al., 2016). Studies on the molecular dissection of seed shattering in domesticated rice have identified different QTL such as *sh3*, *sh4*, and *sh8* (Li et al., 2006; Vigueira et al., 2013).

### Wild Oat

Wild oat (especially *Avena fatua*) is a widespread and competitive weed with a staggered germination pattern, making it a troublesome weed in major winter cereals in many parts of the world (Bullied et al., 2003; Beckie et al., 2012). Wild oats show high levels of seed shattering (Barroso et al., 2006), and seed can remain viable in the soil for up to 18 years (Gonzalez-Andujar and Perry, 1995). The extent of shattering could differ among different *Avena* spp. Bervillé et al. (2005) found that in *A. fatua* the abscission layer forms at the base of individual florets whereas in *A. sterilis*, the layer is developed at the base of an entire spikelet, leading to differences in shattering levels. Mahajan and Chauhan (2021) reported shattering differences between the two species in Queensland, Australia. When localized accessions of wild and cultivated *Avena* spp. are grown together, wild oat seeds matured faster than the cultivated crops and shattered before crop harvest (Maxwell et al., 2007). Seed shattering in wild oat appears to widely vary across geographical locations (Metz, 1969; Wilson, 1970; Feldman and Reed, 1974; Wilson and Cussans, 1975). For example, wild oat seed shattering prior to wheat harvest was reported to be at 22–20% in Saskatchewan, Canada (Burton et al., 2017), and even at 99% in the United Kingdom (Wilson, 1970). Shirtliffe et al. (2000, 2002) indicated that seed shattering pattern in wild oat can be predicted using thermal time, which can inform suitable harvest timing to maximize wild oat seed capture at harvest for subsequent destruction.

### Wild Sunflower

Wild sunflower (*Helianthus annuus*) phenotypically resembles cultivated sunflower, but with a high potential for seed shattering and dispersal (Burke et al., 2002). Shattering in wild sunflower is augmented by the convex floral disc (i.e., high depth:width ratio) due to continued growth of the capitulum. The non-shattering crop types, in contrast, have a relatively flat head (i.e., low depth:width ratio) at maturity (Burke et al., 2002). A considerable density of volunteer sunflower plants can be found in sunflower production fields due to the presence of shattering in cultivated types, leading to yield loss (Reagon and Snow, 2006). The volunteers may arise from the shattered seeds from the same field or nearby fields, leading to competition with the cash crop and significant crop yield loss. Crop volunteers that are commonly found at field edges, alleys, etc. due to unaccounted seed shattering represent a possible channel for gene flow between the cultivated and the common wild sunflower (Massinga et al., 2003; Reagon and Snow, 2006).

## Implications of Seed Shattering

### Crop Improvement

Seed shattering is a detrimental trait in domesticated crops and consistent efforts have been made to eliminate this trait in breeding lines. Advances in molecular technologies have allowed us to develop an improved understanding of the genetic control of this trait in different crop species (Tables 4, 5). The identification of major QTL controlling seed shattering facilitates marker assisted selection (MAS) for improved crop lines with less shattering potential. For instance, EST-SSRs (expressed sequence tag-derived simple sequence repeats) were utilized in breeding for shattering tolerance in wild rye (*Elymus nutans*) (Zhao et al., 2019). The seed shattering-related genes identified in *Arabidopsis* and their orthologs in monocot species could be harnessed for reducing shattering potential (Dong and Wang, 2015). This approach has been utilized in a number of *Brassica* crops (Chandler et al., 2005; Østergaard et al., 2006; Kord et al., 2015). The successful expression of *Arabidopsis* genes in oilseed rape (*Brassica juncea*.) could be attributed to the similar genetic network governing the development of silique valve margin in both species (Østergaard et al., 2006), which remain highly conserved during evolution (Martinez-Anduijar et al., 2012).

Apart from the MAS-based approach, opportunities also exist for introducing shattering tolerance through gene editing/transgenic means. In this context, targeted gene editing technologies, particularly type II Clustered Regularly Interspaced Short Palindromic Repeat (CRISPR)/CRISPR-associated protein 9 (Cas9) could be a potential functional genomics approach for knockdown of gene(s) governing seed shattering in crop plants (Bortesi and Fischer, 2015). A proof of concept for CRISPR-based gene editing for knocking down the *ALCATRAZ* (*ALC*) gene involved in valve margin development has been demonstrated in canola (*Brassica napus*) (Braatz et al., 2017). They transformed the tetraploid oilseed rape (*Brassica napus*) with a CRISPR-Cas9 construct targeting two *ALC* homoeologs and created a transgenic T1 plant with four *alc* mutant alleles. They did not find any wild-type alleles in the T2 generation and all the mutations were stably inherited from T1 to the T2 progeny, which proved that the T1 was a non-chimeric double heterozygote. These promising results indicate that precise nucleotide changes in genes encoding for abscission zone development and valve margin lignification could improve seed and pod shattering resistance in crop plants.

### Crop Management

In crop species that lack a distinct non-shattering system, a number of agronomic and physiological interventions were tested and practiced for reducing seed shattering. In cultivated crops, the adjustment of harvest time based on seed moisture content and the development of abscission zone in reproductive parts is a primary approach to reduce grain yield loss (Silberstein et al., 2010; Shaheb et al., 2015; Xangsayasane et al., 2019). In several species such as *Festulolium*, adhesive preparations or film forming agents applied at the milk-ripe stage when seed moisture content is not less than 60–65% significantly reduced seed shattering (Obraztsov et al., 2018). Cutting seedheads before harvest maturity and allowing them to dry before threshing is another tactic to reduce seed shattering in a number of species such as oilseed *Camelina* (Sintim et al., 2016). Sweating, a variant of swathing, is the practice of placing freshly cut seedheads of grasses in heaps or under a cover for about 3 days before threshing the seeds in order to reduce seed shattering in the field (Hopkinson et al., 2003).

Various chemicals or hormones have been used in several species to reduce seed shattering. For example, *Ascophyllum nodosum* based biostimulant (Sealicit) has been shown to reduce pod shattering and yield loss in oilseed rape (Łangowski et al., 2019). In soybean, plant hormones such as gibberellic acid and nutrient complexes are reported to lower seed shattering (Gulluoglu et al., 2006). However, paclobutrazol, a known antagonist of the plant hormone gibberellin, is reported to improve seed yield in sesame, in part by reducing seed shattering (Mehmood et al., 2021). In birdsfoot trefoil, the use of desiccant sprays (di-n-butyl phthalate, pentachlorophenol, and endothal) were shown to reduce seed shattering (Wiggans et al., 1956).

### Weed Management

The tendency of weed species to either shatter or retain their seeds until the harvest of crops that they infest has great implications for weed population dynamics and management. It is speculated that many weed species, especially in grain crops, have evolved high seed retention potential at crop harvest, which facilitates seed dispersal by harvest machinery and contamination with grain. However, a suite of technologies, collectively known as harvest weed seed control (HWSC) were developed in Australia to capture weed seed at harvest and destroy them, minimizing their dispersal into the field (Walsh et al., 2018). This way, a weed’s ability to retain a high amount of seeds at crop harvest for facilitated dispersal is utilized against them by preventing the seeds from entering the soil seedbank. The efficacy of this system is directly related to the proportion of seeds retained at crop harvest. Significant variations are observed across weed species, cropping systems and climates regarding weed seed retention levels; sowing time adjustment and early-maturing cultivars may facilitate more success with HWSC (Walsh et al., 2018). The agronomic, physiological, hormonal and chemical interventions described above (section “Crop Management”) can be utilized for manipulating seed shattering phenology in weeds and improving seed retention at harvest. However, weeds can evolve to escape HWSC tactics. Ashworth et al. (2016) showed, via recurrent selection, that *Raphanus raphanistrum* (wild radish) has the potential to exhibit early maturity in order to avoid harvest time weed management operations. Sun et al. (2021) further evaluated the early-flowering biotype selected by Ashworth et al. (2016) and confirmed that plants with the early-flowering phenotype retain more pods below the typical wheat harvest height.

Additionally, there are opportunities to employ genetic tools to reduce seed shattering in some of the most problematic weeds with higher seed shattering rates. For example, Yan et al. (2017) proposed a novel approach to partially silence the expression of the seed-shattering gene *SH4* using artificial micro RNA and antisense RNA techniques in weedy rice. However, research efforts in this regard are very limited.

## Conclusion and Future Research Needs

The productivity and economic gains in most food crops are assessed by their seed/grain yield. Besides the genetic potential of a crop to produce a high number of seeds, retention of the seed after physiological maturity till harvest is extremely important. Therefore, consistent breeding efforts have been made to minimize seed shattering in cultivated crops. However, this unique biological trait is highly prevalent in most weed species. Human-driven manipulations have minimized seed shattering in food crops, but is still present at a significant level in many crop species. Knowledge on the physiological and genetic control of seed shattering in plants is useful not only for successful weed management, but also for crop improvement. Yet, there are several unexplored aspects of this important plant trait, especially in an agricultural context. Future research should endeavor to better understand the ecology, physiology and genetics of seed shattering. In particular, seed shattering potential of various agronomically important weed species and the influence of different environmental factors need more research attention. This knowledge will help design and sustain innovative HWSC strategies. Further, potential changes to seed shattering patterns as influenced by adaptive evolution under various management and climate change scenarios warrant adequate investigation.

## Author Contributions

MB conceived the manuscript. AM, AL, and DJ wrote the first draft of the manuscript. All authors edited and revised the manuscript.

## Conflict of Interest

The authors declare that the research was conducted in the absence of any commercial or financial relationships that could be construed as a potential conflict of interest.
